# Small Object Localization with 90% Annotation Reduction by Positive-Unlabeled Learning

**DOI:** 10.3390/mi16121379

**Published:** 2025-12-03

**Authors:** Xiao Zhou, Shihong Wang, Weiguo Hu, Zhaohao Xie, Zheng Pang, Zhuo Jiang, Zhen Cheng

**Affiliations:** 1 Department of Automation, Tsinghua University, Beijing 100084, China; 2 School of Biomedical Engineering, Tsinghua University, Beijing 100084, China; 3 National Engineering Research Center for Beijing Biochip Technology, Beijing 102206, China; 4 College of Food Science, South China Agricultural University, Guangzhou 510642, China; jiangzhuo@scau.edu.cn

**Keywords:** small object, localization, positive-unlabeled (PU) learning, single cell, point annotations

## Abstract

Small object localization is one of the most challenging tasks owing to the poor visual appearance and noisy representation caused by the intrinsic structure of small targets. Recent advances in localizing small objects are mainly dependent on regression-based counting approaches, which require considerable annotations for training. As a contrast, human learners can quickly master labeling skills from only a few annotation examples. In this paper, we attempt to simulate this training mechanism and propose a novel positive-unlabeled (PU) learning based approach that can localize small objects by learning from partial point annotations. We evaluate our approach on five typical datasets of small objects involving a single cell, an animal/insect, and human crowds. Quantitative experimental results show that our approach has achieved inspiring localization performance (F1 score > 0.75) even under the supervision of less than 10% of the overall point annotations. This approach paves the way for low-annotation-cost single-cell analysis within microfluidic droplets.

## 1. Introduction

With the rise of deep convolutional neural networks (CNNs), object detection has shown remarkable progress in the last decade. Localizing small objects, such as cell nuclei in histopathological images [1,2,3,4,5], cells in microfluidic droplets [6,7,8,9], animal/insect populations [10], people in crowds [11,12,13], etc., provides essential clues for the subsequent tasks of segmentation, tracking, density, and morphological analysis. Recent studies of small-object localization mainly focused on predicting a density map of targets [14] and then searching for local density maxima [15,16]. Despite significant advances in the localization of small objects, challenges arise in obtaining adequate center-point annotations for model training in the case of dense-packing small objects, e.g., countless cells in histopathology images or within dense microdroplets [6,7]. Therefore, there is a great need to develop an algorithm that can learn from a spot of point annotation examples and eventually localize and count small objects.

**Figure 1:**
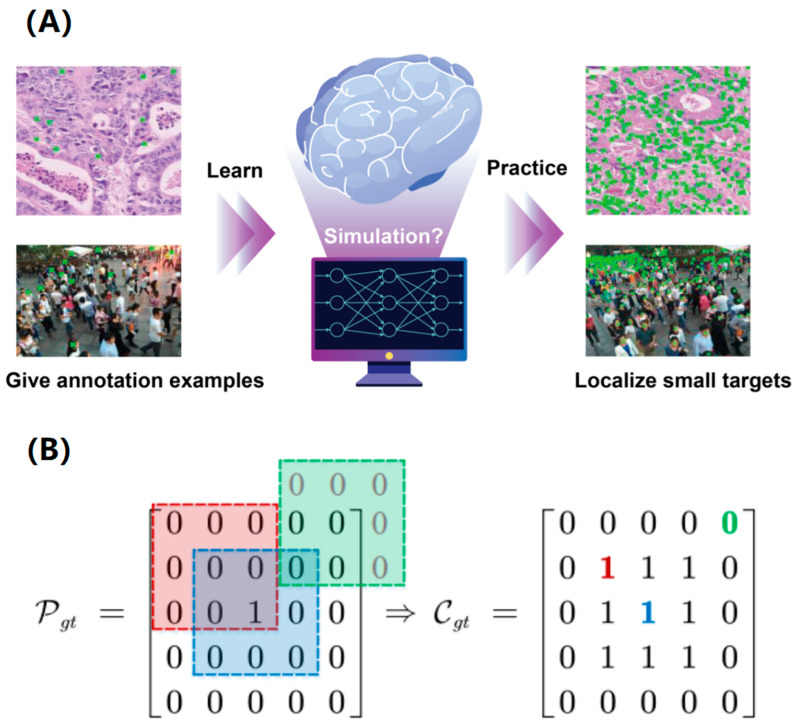
Study motivation and the proposed minimal “redundant count map”. (**A**) The network was designed to learn and localize small objects with limited annotation examples. Green crosses indicate the center of each target. (**B**) Illustrations of the localization map and its corresponding minimal “redundant count map”. The different colors correspond to different 3 × 3 square-kernel positions (left) and their “redundant count map” (right).

**Methodological overview.** Consider a scenario where an experienced individual guides a novice in the task of labeling small objects within a visual image. Typically, the educator only needs to identify a select few exemplary instances for the learner, allowing the learner to perform the annotation task skillfully in accordance with their existing domain-specific knowledge and the examples provided, as illustrated in Figure 1. Inspired by this efficient training mechanism, we expect an algorithm to simulate the learning behavior and then complete the labor-intensive tasks [5,17]. Specifically, the algorithm is supposed to master the skill of localizing small objects by learning from a few point annotations in each training image.

Traditionally, if we treat small object localization as a binary pixel-wise classification problem, each point annotation of the target is viewed as a positive sample while the rest of the region is viewed as a negative background [18]. The pixel-wise classification could hardly function in the case of extremely imbalanced samples between sparse positive points and negative backgrounds. Thereafter, most studies had regressed a continuous density map and managed to localize small targets [11,15]. To predict the density map, regression-based approaches need to previously generate a pseudo-density map as the training ground truth according to the locations of all instances. However, for label reduction, only partial small objects in a training image are provided with point annotations, while the rest of the image remains unknown. In other words, it is improbable to yield an intact and reasonable pseudo-density map from annotated examples. Given that only positive point annotations and unlabeled regions are provided, the positive-unlabeled (PU) learning approach [19] can be intuitively employed to address this tricky problem.

**Key contributions and applications.** We propose a learn-from-educator (LFE) network based on PU learning to tackle the pixel-wise binary classification problem of small object localization. Instead of yielding a continuous density map, we first develop a minimal “redundant count map” that can be generated from incomplete point annotations (see Supplementary Note S1 and Table S1). In addition, the count map greatly alleviates the imbalance between positive targets and a negative background, which allows the PU learning strategy to work effectively in this study. More importantly, the training procedure supervised by the minimal “redundant count map” tends to produce prominent local maxima at the center of cells. Unlike traditional PU learning, which attempts to identify negative samples from the unlabeled data, we introduce an unsupervised label noise modeling approach [20] that can discerningly divide the unlabeled region into positive instances and negative background. Subsequently, supervised by the provided and predicted labels, an ordinary positive–negative learning process is adopted to further improve localization performance. The LFE algorithm is evaluated exhaustively on five different datasets involving cell, animal/insect, and human crowds. We envision that this work also provides an efficient solution for cell-encapsulated microfluidic droplets [6,7], opening a new window for microfluidic applications with minimal annotation cost. The main contributions of this study involves the following:

• We develop a minimal “redundant count map” that can handle the incomplete annotations and tends to produce prominent local maxima at the center of each target in the corresponding predicted location map.
• We introduce an unsupervised loss modeling method to the PU learning strategy, which successfully learns to localize small objects from a few point annotation examples.
• Quantitative experimental results show that our approach can achieve encouraging localization performance even under the supervision of one-tenth of the overall annotations.

## 2. Related Work

**Small object localization.** Instead of time-consuming bounding boxes or mask annotations for object detection or segmentation, a simplified strategy with point annotations is adopted by most object localization studies. These point annotations centered on each object are then treated as density maps, with each annotated location represented by a Gaussian kernel. Early studies, e.g., SC-CNN [1] and SR-CNN [21], employ a traditional regression model to predict the density map of animal populations, in which local maxima suggest instance locations [10,22]. Zhang et al. design a multi-column CNN to regress the density map [23]. Later, many studies [24,25,26] attempt to develop more sophisticated architectures to reduce counting errors. Idrees et al. [15] exploit multiple Gaussian kernels to construct a composition loss to sharpen blurred maps and obtain discrete target positions. Liu et al. [11] adopt a normalized variant of cross-entropy loss to improve localization accuracy. Ma et al. [13] propose a Bayesian loss and manage to acquire remarkable results on the localization. Sam et al. [18] introduce a detection framework to simultaneously predict the localization and size of each object. Zhou et al. propose the SFCN-OPI, which employs a dual-branch structure with target prior interaction to enhance both the detection accuracy and fine-grained classification [16]. Additionally, many biomedical studies [27,28,29,30] are devoted to automatically localizing single cells in histopathological images since the manual annotation by seasoned pathologists is both labor-intensive and costly. He et al. [3] employ multiscale density maps as the ground truth to provide reverse guidance to feature extraction. In order to further reduce annotation cost, weakly supervised (e.g., WSL-LIR [17]) and unsupervised cell detection [2] (e.g., SSAE [31]) are also proposed.

**Positive-unlabeled (PU) learning.** Widely applied to retrieval and outlier detection, PU learning is mainly utilized to train a binary classifier from only positive and unlabeled data [32,33]. The current PU learning approaches can be basically categorized into two branches according to the treatment of unlabeled data. One is to heuristically discriminate negative samples with high confidence from the unlabeled data and then conduct a normal positive–negative learning with the provided positive samples [34]. The other is to directly regard the unlabeled data as negative samples, which suffers from the noise of wrong labels [35]. Aiming to handle a large unlabeled dataset, Sansone et al. [36] propose a scalable PU learning algorithm. Kiryo et al. [19] develop a non-negative risk estimator to avoid the overfitting issue when applying PU learning in sophisticated models, such as neural networks. Instead of recognizing negatives from the unlabeled data, Wang et al. [37] leverage an adaptively augmented algorithm to select positive samples during the training process. Inspired by this approach, we introduce an unsupervised label noise modeling method [20] that characterizes the unlabeled negative loss as a beta mixture model (BMM) with two components to provide pseudo-labels for the unlabeled data. Compared to existing approaches, as shown in Supplementary Table S2, our LFE framework differs fundamentally: it does not rely on full supervision or density map regression, but instead learns effectively from very sparse point annotations via a novel PU-learning mechanism combined with a “redundant count map” representation.

## 3. Methods and Materials

### 3.1. Minimal “Redundant Count Map”

**Figure 2:**
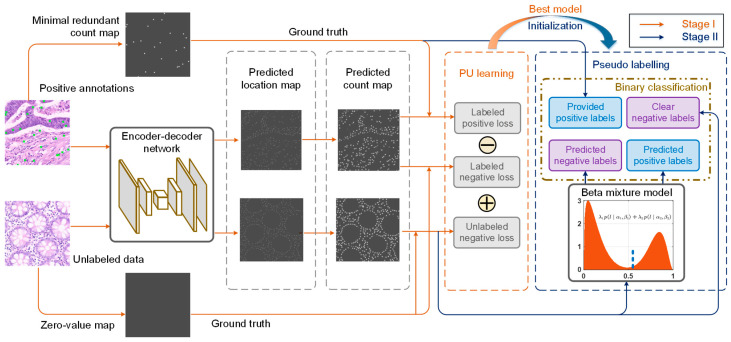
Network architecture and training procedure of the proposed method for small object localization. In the first stage, both positive examples and unlabeled data are first fed into an encoder-decoder network, which outputs predicted location maps of small objects. Afterward, a predicted redundant count map is generated from the predicted location map and then shapes the loss function for the PU learning process. Green crosses indicate the center of each annotation. In the second stage, pseudo-labeling is initialized by the best model in the PU learning procedure. During the pseudo-labeling stage, the negative loss of the unlabeled region in the training images is fitted as a beta mixture model (BMM), and pseudo-labels are produced to perform ordinary binary classification.

Our approach consists of two main stages: PU learning and pseudo-labeling, as illustrated in Figure 2. Before it, in this study, the pseudo-density map utilized in traditional counting-based methods cannot be accurately constructed due to the limited availability of point annotations (only partial targets) within an image. Therefore, we have developed a minimal “redundant count map” that is discretely distributed and, consequently, is able to accommodate incomplete annotations. More importantly, the count map is designed to exhibit a prominent local maximum at each target center.

The method of “redundant count map” was previously proposed in Count-ception [38], wherein a 32 × 32 square kernel was utilized to compute the cell population redundantly. Count-ception is a fully supervised learning approach that regresses a count map whose sum yields the object count. Despite promising counting performance, it fails to localize or predict the *x*, *y* coordinates of each object. This is primarily because it directly predicts the count map, which obscures the precise location of each target. In contrast, our approach predicts a location map, as shown in Supplementary Figure S1.

Assuming that the true location map of all small objects is denoted by *P_gt_*, then each element in the redundant count map *C_gt_* can be computed by summing the pixels within a surrounding square kernel centered at the corresponding location:

(1) $$C_{gt}^{i,j}=\sum_{\left[m,n\right]\;\in \;S_{r}^{i,j}}P_{gt}^{m,n}$$

where $S_{r}^{i,j}$ denotes a square kernel of *r* × *r* centered at the point [*i*, *j*]. Similarly, for the predicted output location map $P_{pre}$, Equation (1) is applied to derive a predicted count map $C_{pre}$.

The “redundant count map”, generated by an *r* × *r* square kernel, offers a discrete alternative to continuous density maps. This eases segmentation into target and unlabeled regions, and boosts the positive pixel rate by a factor of *r* × *r*, effectively alleviating class imbalance for PU learning.

Although a large kernel can significantly increase the positive rate, it may encompass more than one instance in a location map, thereby leading to localization ambiguity, as detailed in Supplementary Note S2. As a compromise, the kernel can be slightly smaller than the minimal size of instances, which not only alleviates the class imbalance but also reduces the localization ambiguity.

It is noteworthy that the redundant count map still preserves the location of each target, which is conducive to generating a prominent local maximum at the target center in the predicted location map, as shown in Supplementary Figure S2. Without loss of generality, assuming that an image with a resolution of 5 × 5 contains a target located at its center and a kernel size of 3 × 3, the location map $P_{gt}$ and its corresponding count map $C_{gt}$ can be depicted as shown in Figure 1B. The L2 loss between the predicted location map $P_{pre}$ and ground truth $C_{gt}$ can be mathematically expressed as follows:

(2) $$\underset{P_{\mathrm{pre}}}{\min}\sum_{i,j}{\left(\sum_{\left[m,n\right]\;\in \;S_{3}^{i,j}}P_{pre}^{m,n}-P_{gt}^{i,j}\right)}^{2}\;s.t.\;P_{pre}^{m,n}\;\geq 0$$

Since ReLU [39] is utilized as the activation function for the network employed in this research, it ensures that the elements in the predicted location map remain non-negative. Accordingly, for those zero-value elements $C_{gt}^{i,j}=0$, the optimal solution for the following equation equals:

(3) $$\{P_{pre}^{m,n}|\left[m,n\right]\;\in \;S_{3}^{i,j}\}\;\overset{yields}{\to }\;\{P_{pre}^{m,n}=0\;|\left[m,n\right]\;\in \;S_{3}^{i,j}\}\;$$

Subsequently, the sparse and precise solution can be generated by setting $P_{pre}^{3,3}=1$, which is equivalent to the location map $P_{gt}$. In other words, the true location map is exactly the optimal solution to Equation (2). Consequently, in the predicted location map, a response distribution with a maximum center will emerge around the location of each true target. Instead of cross-entropy classification loss, L2 loss is utilized to preserve target quantification information, ensuring the predicted location map’s integral reflects the total object count. Since prominent local maxima tend to generate at the instance center in the location map, heuristically identifying local maxima in $P_{pre}$ can provide the precise location of each target.

### 3.2. Positive-Unlabeled (PU) Learning

PU learning is generally used to address the binary classification tasks in which only positive and unlabeled samples are provided. Let *f* be an arbitrary classifier and $\pi_{p}$ be the class-prior probability of positive samples. The empirical risk associated with PU learning can be expressed as follows:

(4) $${\tilde{R}}_{pu}\left(f\right)=\pi_{p}{\hat{R}}_{p}^{+}\left(f\right)\;-\;\pi_{p}{\hat{R}}_{p}^{-}\left(f\right)\;+\;{\hat{R}}_{u}^{-}\left(f\right)$$

where ${\hat{R}}_{p}^{+}\left(f\right)$ denotes the mean empirical risk of positive samples. ${\hat{R}}_{p}^{-}\left(f\right)$ and ${\hat{R}}_{u}^{-}\left(f\right)$ represent the mean empirical risk of assigning positive or unlabeled samples to negative classes, respectively. To address the overfitting issue in PU learning, Kiryo et al. [19] proposed a non-negative risk estimator, which resulted in the re-expression of Equation (4):

(5) $${\hat{R}}_{pu}\left(f\right)=\pi_{p}{\hat{R}}_{p}^{+}\left(f\right)\;+\max\;\left\{0,{\hat{R}}_{u}^{-}\left(f\right)-\;\pi_{p}{\hat{R}}_{p}^{-}\left(f\right)\right\}$$

The loss function in this study is described by this non-negative risk estimator for the PU learning stage, as illustrated in Figure 2. Here, ${\tilde{P}}_{gt}$ and ${\tilde{C}}_{gt}$ are used to represent the partially annotated location map and its corresponding redundant count map, respectively. To implement the PU learning framework, the elements in ${\tilde{C}}_{gt}$ are classified into two categories: non-zero and zero entries. Specifically, the non-zero entries $\Phi_{p}=\left\{\;[i,j]|{\tilde{C}}_{gt}^{i,j}\neq 0\right\}$ can potentially be considered as positive samples. The zero elements $\Phi_{u}=\left\{\;[i,j]|{\tilde{C}}_{gt}^{i,j}=0\right\}$ that cover both positive targets and negative background can be considered as unlabeled data. If we were to directly classify these zero elements $\Phi_{u}$ in ${\tilde{C}}_{gt}$ as unlabeled data, the positive rate in the unlabeled data would be inconsistent with that in the entire training dataset, as it would vary with the number of non-zero entries $\Phi_{p}$ in ${\tilde{C}}_{gt}$. Fortunately, the empirical risks in both Equations (4) and (5) do not require the independence of positive and unlabeled data. Therefore, a straightforward and reliable approach is to utilize the entire training dataset as the unlabeled data.

As previously discussed, the empirical risk is calculated by utilizing the L2 loss function between the predicted count map $C_{pre}$ and ground truth ${\tilde{C}}_{gt}$. Each item of the empirical risk formula in Equation (5) can be determined as follows:

(6) $${\hat{R}}_{p}^{+}\left(f\right)=\;\frac{1}{N_{p}}{\left\|\left(C_{pre}-\;{\tilde{C}}_{gt}\right)⨀M_{gt}\right\|}_{F}^{2}$$

(7) $${\hat{R}}_{u}^{-}\left(f\right)-\pi_{p}{\hat{R}}_{p}^{-}\left(f\right)=\frac{1}{N}{\left\|C_{pre}\right\|}_{F}^{2}-\frac{\pi_{p}}{N_{p}}{\left\|C_{pre}⨀\;M_{gt}\right\|}_{F}^{2}$$

where $C_{pre}^{i,j}=\sum_{\left[m,n\right]\;\in \;S_{r}^{i,j}}P_{pre}^{m,n}$. $M_{gt}$ is a binary indicator mask that denotes the presence of a non-zero value in ${\tilde{C}}_{gt}$. Therefore, the overall sum of $M_{gt}$, symbolically referred to as $N_{p}$, indicates the number of positive samples. The Hadamard product is denoted by ⊙. The total number of entries in $C_{pre}$ is represented by *N*. In our experiments, we discovered that the hyperbolic tangent function exhibits a superior capability in suppressing background noise. Therefore, the negative risk in Equation (7) can be replaced by the following:

(8) $${\hat{R}}_{u}^{-}\left(f\right)-\;\pi_{p}{\hat{R}}_{p}^{-}\left(f\right)=\;\frac{1}{N}\sum_{i,j}\tanh\left(C_{pre}^{i,j}\right)\;-\;\frac{\pi_{p}}{N_{p}}\sum_{i,j}\tanh\left(C_{pre}^{i,j}M_{gt}^{i,j}\right)$$

### 3.3. Pseudo-Labeling

To further improve localization performance, pseudo-labeling is introduced after the PU learning procedure. This technique involves the utilization of pseudo-labels to identify authentic targets with high confidence within the unlabeled region $\Phi_{u}$ of the training images. Subsequently, pseudo-labels are assigned to unlabeled elements present in $\Phi_{u}$, and an ordinary positive–negative learning procedure can be performed. However, care should be taken to judiciously assign false labels to avoid introducing false positives that could adversely affect the training process.

From another perspective, the unlabeled entries $\Phi_{u}$ can also be considered negative samples mixed with some noisy (positive) labels. The goal is to identify these noisy labels and correct them with positive annotations. Thereupon, we compute the element-wise distance between the predicted count map $C_{pre}$ and ground truth ${\tilde{C}}_{gt}$ as the negative loss of the unlabeled entries:

(9) $$l=\left\{{\left|C_{pre}^{i,j}\right|}^{2}|C_{gt}^{i,j}=0\right\}$$

Intuitively, a larger loss in this case suggests a higher probability of noisy labels and vice versa. To adaptively measure this probability, we exploit an unsupervised approach that models the loss distribution of clean and noisy samples by fitting a beta mixture model (BMM) with two components. The probability density function of a BMM on the loss *l* can be given by the following:

(10) $$p(l)=\sum_{k=1}^{K}\lambda_{k}p\;(\left.l\right|\alpha_{k},\beta_{k})$$

where $p\;(\left.l\right|\alpha_{k},\beta_{k})$ denotes a beta distribution and $\alpha_{k},\beta_{k}>0$ denote its parameters. $\lambda_{k}$ represents the mixing weight of the *k*th component. Because the clear negative labels ($l_{i,j}<0.01$) can have a dominant influence on BMM distribution. Therefore, prior to fitting BMM, the clear negative labels are invariably removed. Utilizing an Expectation Maximization (EM) procedure on the loss observations *l*, we can obtain the parameters, i.e., $\alpha_{k},\beta_{k}$ and $\lambda_{k}$ of the BMM. Finally, the probability of an unlabeled entry (corresponding to a local maximum in the predicted location map) being noisy can be computed using the posterior probability:

(11) $$p\left(\left.k=1\right|l_{i,j},\left[i,j\right]\in \psi_{max}\right)$$

where $\psi_{max}$ represents the coordinates set, including all the local maxima found in the predicted location map.

**Figure 3:**
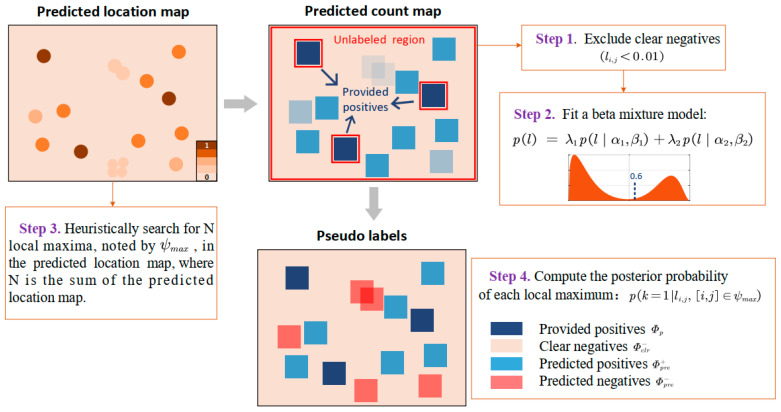
Principle and workflow of the pseudo-labeling procedure. Local maxima in the predicted location map are signified by dots of varying saturation. Squares with different colors were used to represent non-zero count blocks in the corresponding predicted count map.

With the quantification of the confidence of each local maximum being a positive target, a simple confidence threshold can be established to classify $\Phi_{u}$ (unlabeled entries of ${\tilde{C}}_{gt}$) into three categories: clear negatives $\Phi_{clr}^{-}$, predicted positives $\Phi_{pre}^{+}$ and predicted negatives $\Phi_{pre}^{-}$. Combining the provided positives $\Phi_{p}$, a positive–negative learning process can be implemented to further enhance the localization accuracy of small objects, as shown in Figure 3. Given that the kernel size is smaller than the minimum target size, it is plausible to assume that each predicted positive point in the redundant count map covers a single small object. Like the PU learning stage, a L2-*tanh* loss is adopted instead of cross entropy to preserve count information, which can be expressed as follows:

(12) $$L=\;\frac{1}{N}(\sum_{\;[m,n]\in \Phi^{+}}{\left(C_{pre}^{m,n}-1\right)}^{2}\;+\sum_{\;[i,j]\in \Phi^{-}}tanh(C_{pre}^{i,j}))$$

where $C_{pre}^{i,j}=\sum_{\left[m,n\right]\;\in \;S_{r}^{i,j}}P_{pre}^{m,n}.\;\Phi^{-}$ includes the clear and the predicted negatives $\Phi_{clr}^{-}$ and $\Phi_{pre}^{-}$. $\Phi^{+}$ includes the predicted and the provided positives $\Phi_{pre}^{+}$ and $\Phi_{p}$. More details about the pseudo-labeling procedure are shown in Supplementary Note S3.

### 3.4. Architectural Design and Training Details

In architectural design, we have elected to utilize our previous model, LIRNet [17], for the task of predicting the two-dimensional location map of small objects. The authors believe that any encoder-decoder network capable of generating a predicted location map with an identical pixel resolution as the input image will be equally effective. As illustrated in Figure 2, the training is composed of two sequential steps, referred to as PU learning and pseudo-labeling. Both stages are optimized by the Adam optimizer [40] for a total of 100 epochs. The learning rates are initialized with 1 × 10^−4^ in PU learning and 1 × 10^−6^ in pseudo-label learning, respectively, when the batch size is greater than 8. The learning rate is subsequently adjusted based on performance on a separate validation dataset.

During the process of PU learning, it was observed that significant improvements in the localization performance of the network could be achieved by simultaneously incorporating both positive images (few point annotation examples are present in each image) and their enhanced versions (randomly rotated/flipped) into the same training iteration. Consequently, the network is provided with a batch of 4*n* images (where *n* = 1, 2, …), which consists of *n* positive images, *n* enhanced images (generated by randomly transforming the *n* positive images), and 2*n* unlabeled images from a new data loader.

In each training epoch, we randomly select 2/3 of the given point annotations in each training image to yield the ground truth of the location map, which proves to be highly effective in stabilizing the training procedure and preventing overfitting. Considering that searching for local maxima in a predicted location map can be computationally expensive, the early period of PU training may yield unreasonable maps, thus hindering the localization process. To circumvent this issue, a delay of 20 epochs is implemented before initiating the quantification of localization performance. The model that exhibits the best performance on a validation dataset in PU learning is saved and utilized as the initialization for the pseudo-labeling process.

### 3.5. Parameter Configuration

Accurately adjusting two key parameters, including the kernel size *r* in Equation (1) and the class-prior probability of positive samples in Equation (8), is critical when processing different datasets. It is important to note that these two parameters are not independent, meaning that if one is fixed, the other can be estimated. As mentioned earlier, all non-zero entries in the ground truth ${\tilde{C}}_{gt}$ are considered positive samples. The mathematical computation of each entry in ${\tilde{C}}_{gt}$ is calculated by the sum of pixels in a square kernel of *r* × *r* from the corresponding location map.

Consequently, the class-prior probability of positive samples $\pi_{p}$ can be estimated by calculating the average proportion of non-zero entries in a complete count map *C_gt_* (not ${\tilde{C}}_{gt}$, which contains only partial positive annotations). It can be expressed as $\pi_{p}\;\approx \;\frac{\mu r^{2}}{\omega h}$, where μ represents the average number of targets in one image, h and ω represent the height and width of the count map. Assuming that μ can be provided or estimated approximately, then $\pi_{p}$ can be calculated by fixing the kernel size *r*. In order to achieve a compromise between class imbalance and localization ambiguity, *r* is necessary to be slightly smaller than the minimum size of a target in each dataset. To prevent over-tuning of parameters, $\pi_{p}$ is set to 0.1 for all subsequent experiments.

### 3.6. Dataset and Evaluation Metric

To evaluate the effectiveness of the proposed approach, we evaluated it on five representative small object datasets, including two hematoxylin and eosin (H&E) stained histopathology image datasets [1,39], two animal population video sequences [5], and one human crowd dataset [13] named ShanghaiTech Part B (abbreviated as ST Part B). For the two animal datasets, which were captured from video sequences, we utilized only the first 32 images, and the subsequent 8 images were utilized for training and validation, consistent with the previous study [10].

As two recently collected human H&E datasets, the Colorectal Adenocarcinoma (CA) dataset and the Modified Bone Marrow (MBM) dataset, were randomly divided into training, validation, and test datasets. Since the ST Part B dataset was provided with fixed training and test samples, we randomly selected a small portion of the training samples as a validation dataset. The detailed split of each dataset is shown in the Supplementary Note S4 and Table S3. We evaluate the performance of the proposed approach with a varying number of point annotation examples, according to the average number of instances in each dataset, as shown in Table 1. For each number of labeled examples, we generated 10 repeated sample selections, each with a different random seed, to train the localization model. Performance results under different numbers of labeled examples were reported by mean and variance across the 10 repeats.

Several metrics, including accuracy, recall, and F1 score, have been employed to evaluate the localization performance of different approaches. For the two histopathology image datasets, if the distance between a target and its nearest location prediction is less than 6 pixels, the location prediction is considered a true positive (TP), consistent with previous studies [1,17]. For the two small animal datasets, the distance threshold for distinguishing a TP is set to the minimal object radius (12 and 5 pixels for Honeybee and Fish, respectively), consistent with the previous study [10]. For the ST Part B dataset, we adopt the literature’s metric Mean Localization Error (MLE) [41] to measure the localization performance between the predicted locations and their matched ground truth.

## 4. Results and Discussion

### 4.1. Prominent Local Maxima for Object Localization

To verify the hypothesis that the proposed minimal “redundant count map” tends to generate a prominent local maximum at each target center, we extract the pixel value of each predicted target and its proportion in a square kernel in the predicted location map. Experimental results of prominent local maxima for each dataset are summarized in Table 2. The kernel size *r* was supposed to cover 99.7% of instances following a normalized Gaussian distribution, i.e., $\frac{r}{2}=3\sigma$. Therefore, the Gaussian center in Table 2 represents the central response of a normalized Gaussian distribution with a standard deviation of $\sigma =\frac{r}{6}$. It is found that although the mean local maximum and its mean proportion over the kernel in the predicted location maps decrease as the target/kernel size increases, they are still higher than the Gaussian center by a large margin (greater than three times), especially for Honeybee, Fish, MBM, and ST Part B datasets.

**Figure 4:**
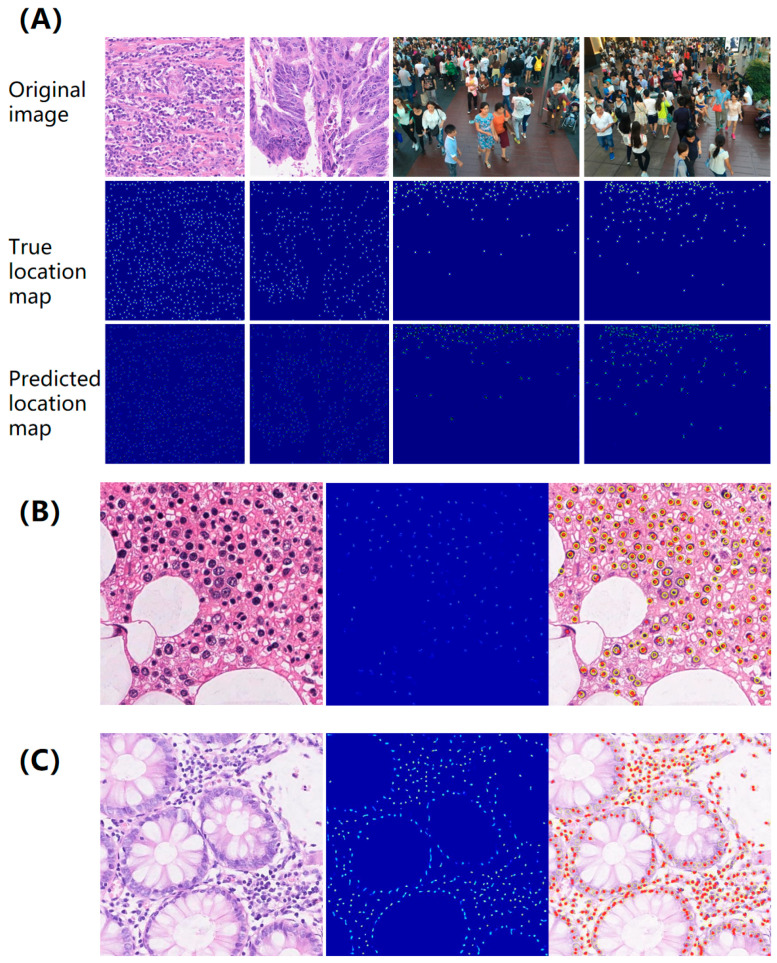
Representative predicted location maps in instance-dense regions. (**A**) From top to bottom: original image, ground-truth location map, and predicted location map. (**B**) Typical results of the LFE framework for nuclei detection on the Colorectal Adenocarcinoma (CA) cell datasets. (**C**) Typical results of the LFE framework for nuclei detection on the Modified Bone Marrow (MBM) cell datasets. The yellow circles and the red dots correspond to ground-truth and the predicted centers, respectively. For optimal clarity, please refer to the color and zoomed versions of the figures.

The results of the true and the predicted location map are presented in Figure 4, which illustrates typical examples of location maps in instance-dense regions. It is observed that each target is visually independent even in dense crowds, indicating the effectiveness of the proposed map in handling incomplete annotations. In addition, a key advantage of this method is its tendency to yield prominent local maxima at the centers of targets, serving as reliable markers for localizing small objects (see Supplementary Figure S2). Additionally, the uniqueness of the proposed solution to the minimization problem is separately validated through a theoretical derivation provided in Supplementary Note S5. It ensures that our model converges to a single, plausible estimate of the underlying object locations. This property, enhanced by our well-conditioned transformation, makes the learning process inherently robust to noise and ambiguities in the training annotations. In conclusion, the proposed minimal “redundant count map” is evaluated for localized instances with incomplete annotations and a tendency toward prominent local maxima at the object center.

### 4.2. Performance Comparison Between Baseline and the Proposed Method

**Figure 5:**
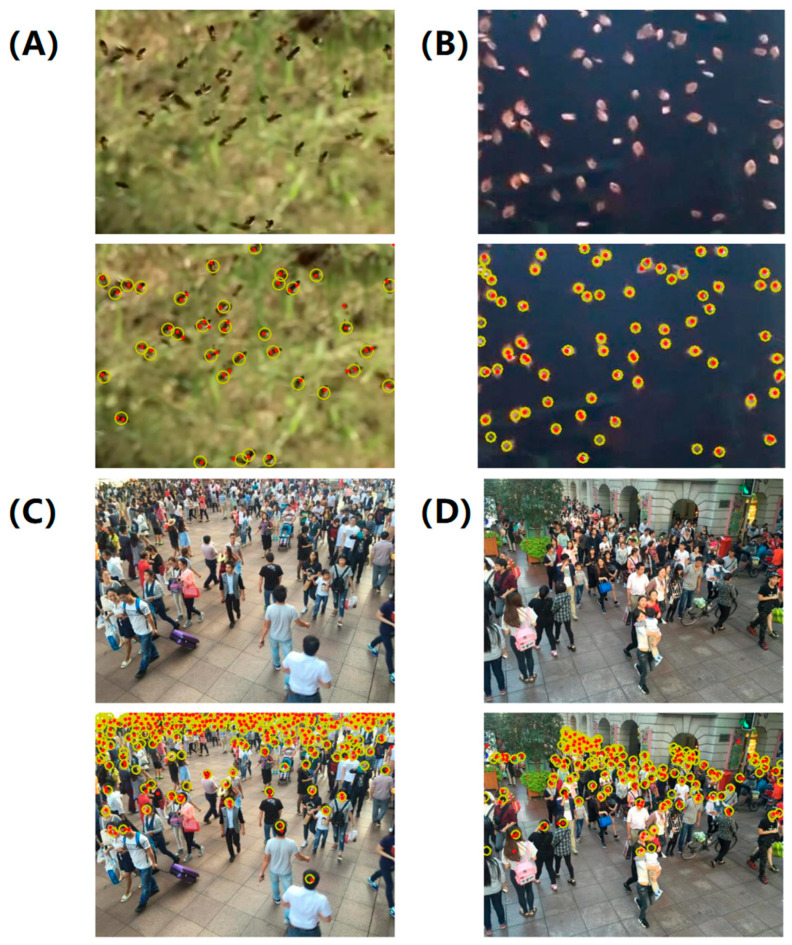
Representative localization results on test datasets. Models were trained with fewer than 10% point annotations: (**A**) honeybee; (**B**) bish; (**C**) and (**D**) human crowd. Yellow circles and red dots denote the ground-truth and predicted object centers, respectively.

We further quantify the localization performance of the proposed approach and compare it with baseline methods. Experimental results on each test dataset are shown in Table 3, Table 4, Table 5, Table 6 and Table 7 with a varying number of point annotations, in which LFE represents our proposed approach, and the following digits represent the number of point annotations employed in the training process. For example, LFE-3 indicates that only three annotations were provided in each image for training LFE. The percentage of labels in the last column also shows the proportion of partially annotated examples. The bold black font indicates the best performance, and the blue font highlights the performance of the proposed LFE supervised by approximately 10% of all annotation points. The representative visualization of localization results supervised by less than 10% of point annotations is also shown in Figure 5.

**CA cells dataset.** Table 3 provides a comparison of the proposed approach’s localization performance against other sophisticated methods, including SSAE [31], SR-CNN [21], SC-CNN [1], SFCN-OPI [16], SSL-LIR, and WSL-LIR [17], on the CA cell test dataset. The results indicate that our LFE method utilizing 100 and 50 labeled examples achieves comparable performance to fully supervised SC-CNN and SR-CNN approaches, despite being supervised by only 33.7% and 16.8% of the annotated points, respectively. Furthermore, when compared to our previous weakly supervised WSL-LIR [17], the LFE-50 can reduce annotation costs by more than 50%, while maintaining an almost identical F1 score. It is also concluded that the PU learning strategy with unsupervised loss modeling can successfully learn to localize small instances from a few point annotation examples.

**Table 3:** The localization performance on the CA cells dataset.

Method	Precision	Recall	F1 Score ↑	Labels
SSAE	0.617	0.644	0.630	0%
SR-CNN	0.783	0.804	0.793	100%
SC-CNN	0.781	0.823	0.802	100%
SFCN-OPI	0.819	0.874	0.834	100%
SSL-LIR	0.854	0.850	**0.852**	100%
WSL-LIR	0.810	0.777	0.793	43.8%
LFE-100	0.78 ± 0.02	0.83 ± 0.02	0.80 ± 0.01	33.7%
LFE-50	0.76 ± 0.02	0.82 ± 0.01	0.79 ± 0.01	16.8%
LFE-25	0.75 ± 0.02	0.81 ± 0.02	**0.78 ± 0.01**	**8.4%**
LFE-10	0.74 ± 0.02	0.75 ± 0.04	**0.74 ± 0.02**	**3.4%**

Note: The black bold font indicates the best performance, and the blue with underline highlights the performance of our approach supervised by approximately 10% of all point annotations. “↑” indicates “larger is better.”

Interestingly, when the label reduction is increased by more than 91.6%, the F1 score of the LFE-25 is only 0.023 lower than that of the LFE-100, indicating that our approach is not overly sensitive to label reduction. Even with a further reduction in labeled examples, the LFE-10 (adopting only 3.4% labels) still outperforms the unsupervised SSAE approach by a considerable margin. A distinctive advantage of LFE over unsupervised and fully supervised methodology is its ability to exploit data scarcity. In scenarios where a minute portion (<10%) of the data is labeled, LFE can optimize the existing information by focusing on the most enlightening examples. This strategy can potentially lead to more precise forecasting and enhanced generalization capabilities.

**MBM cells dataset.** Table 4 exhibits the performance quantification of our approach at different levels of label reduction on histopathological bone marrow images. Exploiting less than 39.7% of annotated points, the LFE-50 network achieves comparable localization performance with the fully supervised SSL-LIR approach [17]. In addition, when less than 8% of labeled examples are utilized for training, the LFE-10 can still precisely localize more than 83% of the targets in the test dataset, suggesting its effectiveness. The LFE framework offers several potential benefits over fully supervised approaches (detailed in Supplementary Note S6), while a key benefit is that it allows for a more efficient use of labeled data.

**Table 4:** The localization performance on the MBM cells dataset.

Method	Precision	Recall	F1 Score ↑	Labels %
SSL-LIR	0.885	0.873	**0.879**	100%
LFE-50	0.83 ± 0.02	0.90 ± 0.02	0.87 ± 0.01	39.7%
LFE-25	0.81 ± 0.02	0.88 ± 0.01	0.84 ± 0.01	19.8%
LFE-10	0.76 ± 0.01	0.83 ± 0.04	**0.79 ± 0.01**	**7.9%**
LFE-5	0.75 ± 0.03	0.76 ± 0.03	**0.75 ± 0.02**	**4.0%**

Note: The black bold font indicates the best performance, and the blue with underline highlights the performance of our approach supervised by approximately 10% of all point annotations. “↑” indicates “larger is better.”

**Honeybee and Fish datasets.** Compared to the SIDIP method [10], the LFE-15 model achieves the same localization performance on the Honeybee test dataset, as shown in Table 5, while requiring only 53.6% of labeled points. Because the number of honeybees in each image is relatively small, 90% label reduction means fewer than three targets in an image have been annotated. In this situation, our LFE-3 algorithm still successfully detected about 83% of the small honeybees in the test dataset, while 72% of the location predictions suggest true positives, proving the feasibility of small object localization with 90% annotation reduction.

**Table 5:** The localization performance on the Honeybee dataset.

Method	Precision	Recall	F1 Score ↑	Labels
SIDIP	0.921	0.787	0.849	100%
LFE-15	0.79 ± 0.04	0.91 ± 0.03	**0.85 ± 0.03**	53.6%
LFE-10	0.78 ± 0.04	0.91 ± 0.03	0.84 ± 0.03	35.7%
LFE-5	0.68 ± 0.06	0.86 ± 0.04	0.76 ± 0.03	17.9%
LFE-3	0.72 ± 0.11	0.83 ± 0.04	**0.76 ± 0.05**	**10.7%**

Note: The black bold font indicates the best performance, and the blue underlined text highlights the performance of our approach supervised by approximately 10% of all point annotations. “↑” indicates “larger is better.”

Comparative localization performance on the Fish dataset was also demonstrated in Table 6. It was observed that even though less than 20% of labeled targets were provided for the training stage, our LFE-10 approach still outperforms the fully supervised SIDIP method. In addition, the localization performance of the LFE-5 with 91.1% label reduction is very close to that of SIDIP using 100% annotations. To further demonstrate the effectiveness of the proposed LFE-10 algorithm, we conducted a series of experiments on both datasets. The results are shown in Figure 5A,B, showing that nearly all of the small objects are captured and localized. The LFE-10 algorithm achieves an average localization error of 2 pixels, which is better than the fully supervised SIDIP method. The LFE-10 algorithm also achieved a higher precision and recall rate of 96% and 94% compared to the SIDIP method.

**Table 6:** The localization performance on the Fish dataset.

Method	Precision	Recall	F1 Score ↑	Labels
SIDIP	0.951	0.921	0.936	100%
LFE-25	0.97 ± 0.01	0.95 ± 0.01	**0.96 ± 0.01**	44.6%
LFE-15	0.95 ± 0.02	0.95 ± 0.01	0.95 ± 0.01	26.8%
LFE-10	0.96 ± 0.01	0.94 ± 0.02	0.95 ± 0.01	17.9%
LFE-5	0.94 ± 0.03	0.90 ± 0.05	**0.92 ± 0.04**	**8.9%**

Note: The black bold font indicates the best performance, and the blue with underline highlights the performance of our approach supervised by approximately 10% of all point annotations. “↑” indicates “larger is better.”

**ST Part B.** A detailed study of the ST Part B dataset of human crowds shows that the proposed LFE method outperforms other state-of-the-art methods in the MLE metric. The performance comparison on the ST Part B dataset is exhibited in Table 7, where LS-CNN and CSR-A-thr results are directly from the literature [41]. The proposed LFE supervised by 82.3% of point annotations outperforms the fully supervised LSC-CNN method. Encouragingly, even though only 10% of the point annotations (12 examples) are randomly selected to supervise our training procedure, the average localization error is still lower than that of CSR-A-thr by a large margin. A representative visualization of the localization results was shown in Supplementary Figure S3, suggesting that our method would not deteriorate significantly with the decreasing number of annotation examples. The ability of the proposed method to handle complex data structures and large datasets makes it a superior choice for localization tasks of small objects.

**Table 7:** The localization performance on the ST Part B dataset.

Method	MLE ↓	Labels
CSR-A-thr	12.28	100%
LSC-CNN	9.0	100%
LFE-100	**8.70 ± 0.14**	81.3%
LFE-50	9.63 ± 0.21	40.6%
LFE-25	10.10 ± 0.24	20.3%
LFE-12	**10.79 ± 0.43**	**9.8%**

Note: The black bold font indicates the best performance, and the blue with underline highlights the performance of our approach supervised by approximately 10% of all point annotations. “↓” indicates “smaller is better.”

### 4.3. Ablation Study on Pseudo-Labeling

**Figure 6:**
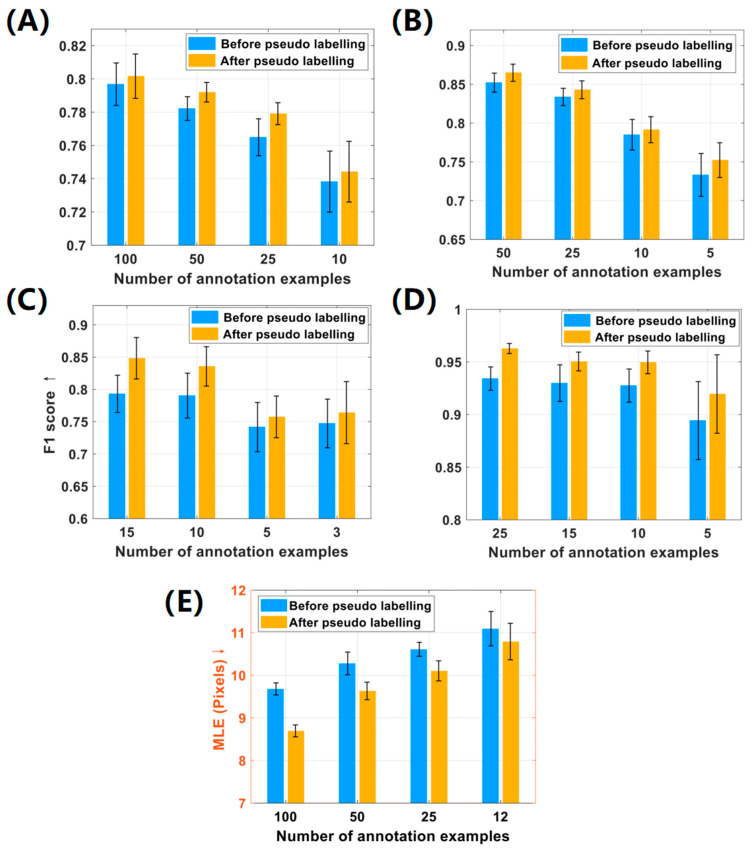
Comparison of localization performance with and without pseudo-labeling. Quantitative results across five datasets: (**A**) CA cells; (**B**) MBM cells; (**C**) honeybee; (**D**) fish; (**E**) human crowd. Performance is evaluated using the F1 score (“↑”, higher is better) and the Mean Localization Error (MLE; “**↓**”, lower is better).

To demonstrate the effectiveness of the pseudo-labeling strategy in the LFE method, we recorded and analyzed the localization performance before and after the pseudo-labeling process. The experimental results are depicted in Figure 6. It is notable that the implementation of pseudo-labeling boosted the overall F1 score of localization by 0.5%~5% in the first four datasets. Specifically, in the Honeybee and Fish datasets, the average performance after pseudo-labeling was notably improved by 3.32% and 2.41%, respectively. In the CA and MBM cell datasets, significant improvements in F1 scores were also noted in different numbers of point annotations.

Similarly, the pseudo-labeling process in the ST Part B dataset significantly reduced the mean localization error MLE by 5.9%. Objectively speaking, the efficiency of the LFE method can deteriorate with the reduction of labeled examples due to the decline in supervised information. This is because algorithms rely on the abundance of data to generalize and make accurate predictions. Without sufficient examples, most algorithms may not be able to fully capture the variations and complexity in the data. However, Figure 6 indicates that the LFE method performs remarkably well on the Fish dataset, even with a label reduction of more than 90%. This is attributed to the fact that the small objects in this dataset share similar morphological features, which are less susceptible to the perspective changes. Furthermore, it is evident from Figure 6 that the standard deviation (black error bar) of the F1 score increases with the reduction of labels, because fewer labeled examples are less likely to cover all visual features of targets compared to a majority of annotations. In summary, our method demonstrated consistent performance across all datasets, showing notable improvement in localization performance after pseudo-labeling. In addition, our method is robust for label reduction, as it can effectively handle datasets with more than 90% unlabeled examples.

## 5. Discussions and Conclusions

Small object localization has found widespread applicability in diverse fields, including single-cell detection, droplet microfluidics, animal population monitoring, and crowd counting. Inspired by the learning behavior exhibited by human annotators, we developed a PU learning algorithm, the learn-from-educator (LFE) model, specifically designed to localize small objects by exploiting a limited number of point-based annotations. Instead of regressing a continuous density map of small objects, LFE employs a discrete minimal “redundant count map”, which tends to produce a prominent local maximum at the spatial center of the target in the predicted location map. To further enhance localization performance, we have incorporated an unsupervised loss modeling approach into the PU learning strategy, which provides pseudo-labels for a subsequent normal positive–negative learning process. If only a small proportion of the data is labeled, LFE may be a more efficient approach (Supplementary Table S4) as it can handle incomplete annotations and make more productive use of available information. Quantitative experimental results suggest that our algorithm significantly reduces annotation costs and achieves encouraging localization performance. The LFE system holds significant potential for application in droplet microfluidics, enabling precise, high-throughput droplet counting and single-cell localization while drastically reducing annotation costs. Looking forward, the straightforward adaptation of our framework to dynamic environments like live-cell microscopy or microfluidics presents a promising direction. By fine-tuning on a minimal annotated subset from a new domain, our data-efficient approach can be readily deployed for temporal analysis.

## Figures and Tables

**Table 1:** The number of point annotations used in different datasets.

Dataset	Number of Targets per Image	Number of Point Annotations
CA cells	28 ± 6	15, 10, 5, 3
MBM cells	56 ± 9	25, 15, 10, 5
Fish	126 ± 33	50, 25, 10, 5
Honeybee	298 ± 217	100, 50, 25, 10
ST Part B	123 ± 94	100, 50, 25, 12

**Table 2:** The quantitative results of local maxima in different datasets.

Dataset	Kernel Size	Local Maximum	Mean Proportion	Gaussian Center
CA	7	0.24 ± 0.06	0.27 ± 0.05	0.12
MBM	11	0.22 ± 0.05	0.23 ± 0.05	0.05
Fish	9	0.20 ± 0.06	0.21 ± 0.06	0.07
Honeybee	15	0.16 ± 0.06	0.19 ± 0.06	0.03
ST Part B	17	0.13 ± 0.02	0.15 ± 0.01	0.02

## Data Availability

The original contributions presented in this study are included in the article and Supplementary Materials. Further inquiries can be directed to the corresponding author. The code is available on GitHub at the following link https://github.com/wangshihong/LFE (accessed on 20 October 2025).

## Supplementary Materials

The following supporting information can be downloaded at https://www.mdpi.com/article/10.3390/mi16121379/s1, Supplementary Note S1. Brief description of localization ambiguity. Note S2. Improve localization performance by pseudo-labelling. Note S3. Detailed illustration for the dataset split. Note S4. Difference between density-map approaches and our method. Note S5. Theoretical derivation for the uniqueness of our method. Note S6. The benefits of the proposed method over fully supervised approaches. Figure S1. Comparison of different annotation and map generation methods. Figure S2. Illustration of the localization ambiguity. Figure S3. Illustration of the optimal solution and the uniqueness of the proposed method. Figure S4. Typical visualization of the localization results on a human crowd using 10% of point annotations. Table S1. The comparison of different losses between density-map- based approaches and our method. Table S2. The summary of different methods that related to this study. Table S3. The structure and split of each dataset used in this paper. Table S4. Benchmark of models: parameters, size, and methodology.

## References

[1] Sirinukunwattana K., Raza S.E.A., Tsang Y.W., Snead D.R.J., Cree I.A., Rajpoot N.M. (2016). Locality Sensitive Deep Learning for Detection and Classification of Nuclei in Routine Colon Cancer Histology Images. IEEE Trans. Med. Imaging.

[2] Hou L., Nguyen V., Kanevsky A.B., Samaras D., Kurc T.M., Zhao T.H., Gupta R.R., Gao Y., Chen W.J., Foran D. (2019). Sparse autoencoder for unsupervised nucleus detection and representation in histopathology images. Pattern Recogn..

[3] He S.H., Minn K.T., Solnica-Krezel L., Anastasio M.A., Li H. (2021). Deeply-supervised density regression for automatic cell counting in microscopy images. Med. Image Anal..

[4] Hagos Y.B., Narayanan P.L., Akarca A.U., Marafioti T., Yuan Y.Y. ConCORDe-Net: Cell Count Regularized Convolutional Neural Network for Cell Detection in Multiplex Immunohistochemistry Images. Proceedings of the Medical Image Computing and Computer Assisted Intervention—MICCAI 2019.

[5] Zhou X., Gu M., Cheng Z. (2021). Local integral regression network for cell nuclei detection. Entropy.

[6] Zhou X., Mao Y., Gu M., Cheng Z. (2023). WSCNet: Biomedical Image Recognition for Cell Encapsulated Microfluidic Droplets. Biosensors.

[7] Mao Y., Zhou X., Hu W., Cheng Z. (2024). Dynamic video recognition for cell encapsulated microfluidic droplets. Analyst.

[8] Mao Y., Zhou X., Hu W., Yang W., Cheng Z. Adaptive sparse pairwise loss for object re-identification. Proceedings of the 2023 IEEE Conference on Computer Vision and Pattern Recognition (CVPR).

[9] Xiong B., Su C., Lin Z., Chen Y., Zhou Y., Cheng Z., Yu Z., Huang T. Real-time parameter evaluation of high-speed microfluidic droplets using continuous spike streams. Proceedings of the 2024 ACM International Conference on Multimedia (MM’ 24).

[10] Ma Z., Yu L., Chan A.B. Small Instance Detection by Integer Programming on Object Density Maps. Proceedings of the 2015 IEEE Conference on Computer Vision and Pattern Recognition (CVPR).

[11] Liu C.C., Weng X.Y., Mu Y.D. Recurrent Attentive Zooming for Joint Crowd Counting and Precise Localization. Proceedings of the IEEE/CVF Conference on Computer Vision and Pattern Recognition (CVPR).

[12] Liu W.Z., Salzmann M., Fua P. Context-Aware Crowd Counting. Proceedings of the IEEE/CVF Conference on Computer Vision and Pattern Recognition (CVPR).

[13] Ma Z.H., Wei X., Hong X.P., Gong Y.H. Bayesian Loss for Crowd Count Estimation with Point Supervision. Proceedings of the 2019 IEEE/CVF International Conference on Computer Vision (ICCV 2019).

[14] Zhou X.Q., Zou Y.X., Wang Y. Accurate Small Object Detection Via Density Map Aided Saliency Estimation. Proceedings of the 2017 IEEE International Conference on Image Processing (ICIP).

[15] Idrees H., Tayyab M., Athrey K., Zhang D., Al-Maadeed S., Rajpoot N., Shah M. Composition Loss for Counting, Density Map Estimation and Localization in Dense Crowds. Proceedings of the Computer Vision—ECCV.

[16] Zhou Y.N., Dou Q., Chen H., Qin J., Heng P.A. SFCN-OPI: Detection and Fine-Grained Classification of Nuclei Using Sibling FCN with Objectness Prior Interaction. Proceedings of the AAAI Conference on Artificial Intelligence.

[17] Zhou X., Cheng Z., Gu M., Chang F. LIRNet: Local Integral Regression Network for Both Strongly and Weakly Supervised Nuclei Detection. Proceedings of the 2020 IEEE International Conference on Bioinformatics and Biomedicine (BIBM).

[18] Sam D.B., Babu R.V. Top-Down Feedback for Crowd Counting Convolutional Neural Network. Proceedings of the AAAI Conference on Artificial Intelligence.

[19] Kiryo R., Niu G., Plessis M.C.d., Sugiyama M. Positive-unlabeled learning with non-negative risk estimator. Proceedings of the 31st International Conference on Neural Information Processing Systems.

[20] Arazo E., Ortego D., Albert P., O’Connor N.E., McGuinness K. Unsupervised Label Noise Modeling and Loss Correction. Proceedings of the International Conference on Machine Learning.

[21] Xie Y.P., Xing F.Y., Kong X.F., Su H., Yang L. (2015). Beyond Classification: Structured Regression for Robust Cell Detection Using Convolutional Neural Network. International Conference on Medical Image Computing and Computer-Assisted Intervention.

[22] Xie Y.P., Xing F.Y., Shi X.S., Kong X.F., Su H., Yang L. (2018). Efficient and robust cell detection: A structured regression approach. Med. Image Anal..

[23] Zhang Y.Y., Zhou D.S., Chen S.Q., Gao S.H., Ma Y. Single-Image Crowd Counting via Multi-Column Convolutional Neural Network. Proceedings of the 2016 IEEE Conference on Computer Vision and Pattern Recognition (CVPR).

[24] Li Y.H., Zhang X.F., Chen D.M. CSRNet: Dilated Convolutional Neural Networks for Understanding the Highly Congested Scenes. Proceedings of the 2018 IEEE/CVF Conference on Computer Vision and Pattern Recognition (CVPR).

[25] Cao X.K., Wang Z.P., Zhao Y.Y., Su F. Scale Aggregation Network for Accurate and Efficient Crowd Counting. Proceedings of the Computer Vision—ECCV.

[26] Jiang X., Xiao Z., Zhang B., Zhen X., Cao X., Doermann D., Shao L. Crowd counting and density estimation by trellis encoder-decoder networks. Proceedings of the IEEE/CVF Conference on Computer Vision and Pattern Recognition.

[27] Zhu R.K., Sui D., Qin H., Hao A.M. An Extended Type Cell Detection and Counting Method based on FCN. Proceedings of the 2017 IEEE 17th International Conference on Bioinformatics and Bioengineering (BIBE).

[28] Khan A., Gould S., Salzmann M. Deep convolutional neural networks for human embryonic cell counting. Proceedings of the European Conference on Computer Vision.

[29] Raza S.E.A., AbdulJabbar K., Jamal-Hanjani M., Veeriah S., Le Quesne J., Swanton C., Yuan Y.Y. Deconvolving Convolutional Neural Network for Cell Detection. Proceedings of the IEEE 16th International Symposium on Biomedical Imaging (ISBI 2019).

[30] Xie W., Noble J.A., Zisserman A. (2018). Microscopy cell counting and detection with fully convolutional regression networks. Comput. Methods Biomech. Biomed. Eng. Imaging Vis..

[31] Xu J., Xiang L., Liu Q.S., Gilmore H., Wu J.Z., Tang J.H., Madabhushi A. (2016). Stacked Sparse Autoencoder (SSAE) for Nuclei Detection on Breast Cancer Histopathology Images. IEEE Trans. Med. Imaging.

[32] Elkan C., Noto K. Learning classifiers from only positive and unlabeled data. Proceedings of the 14th ACM SIGKDD International Conference on Knowledge Discovery and Data Mining.

[33] Hido S., Tsuboi Y., Kashima H., Sugiyama M., Kanamori T. Inlier-based Outlier Detection via Direct Density Ratio Estimation. Proceedings of the 2008 Eighth IEEE International Conference on Data Mining.

[34] Li X., Liu B. Learning to classify texts using positive and unlabeled data. Proceedings of the 18th international joint conference on Artificial intelligence.

[35] Bing L., Yang D., Li X.L., Lee W.S., Yu P.S. Building text classifiers using positive and unlabeled examples. Proceedings of the Third IEEE International Conference on Data Mining.

[36] Sansone E., De Natale F.G.B., Zhou Z.H. (2018). Efficient Training for Positive Unlabeled Learning. IEEE Trans. Pattern Anal. Mach. Intell..

[37] Wang Z., Long G. Positive Unlabeled Learning by Sample Selection and Prototype Refinement. Proceedings of the Advanced Data Mining and Applications.

[38] Cohen J.P., Boucher G., Glastonbury C.A., Lo H.Z., Bengio Y. Count-ception: Counting by Fully Convolutional Redundant Counting. Proceedings of the 2017 IEEE International Conference on Computer Vision Workshops (ICCVW 2017).

[39] Glorot X., Bordes A., Bengio Y. Deep Sparse Rectifier Neural Networks. Proceedings of the 14th International Conference on Artificial Intelligence and Statistics.

[40] Kingma D., Ba J. (2014). Adam: A Method for Stochastic Optimization. arXiv.

[41] Sam D.B., Peri S.V., Sundararaman M.N., Kamath A., Babu R.V. (2020). Locate, Size, and Count: Accurately Resolving People in Dense Crowds via Detection. IEEE Trans. Pattern Anal. Mach. Intell..

