# Supplementary material for: Small Object Localization with 90% Annotation Reduction by Positive-Unlabeled Learning
Source: Micromachines (Basel). 2025 Dec 3;16(12):1379. doi: 10.3390/mi16121379 (PMC12735266; doi:10.3390/mi16121379)
Supplement: Supplementary file 1 [file micromachines-16-01379-s001.zip › micromachines-3974403-supplementary.pdf]

Supplementary Information

Small object localization with 90% annotation reduction by positive-unlabeled learning

Xiao Zhou [a], Shihong Wang [b,d], Weiguo Hu [a], Zhaohao Xie [b,c], Zheng Pang [b,d], Zhuo Jiang [c], Zhen Cheng [a,d] *

[a] Department of Automation, Tsinghua University, Beijing, 100084, China.

[b] School of Biomedical Engineering, Tsinghua University, Beijing, 100084, China.

[c] College of Food Science, South China Agricultural University, Guangzhou Guangdong China 510642, China.

[d] National Engineering Research Center for Beijing Biochip Technology, Beijing, 102206, China.

*Corresponding authors: Zhen Cheng, Department of Automation, Tsinghua University, Haidian District, Beijing, 100084, China.

Phone: +86-10-61777481, fax: +86-10-61777481, email: zcheng@mail.tsinghua.edu.cn.

Table of contents

Supplementary Note S1. Brief description of localization ambiguity.

Supplementary Note S2. Improve localization performance by pseudo-labeling.

Supplementary Note S3. Detailed illustration for the dataset split.

Supplementary Note S4. Difference between density-map approaches and our method.

Supplementary Note S5. Theoretical derivation for the uniqueness of our method.

Supplementary Note S6. The benefits of the proposed method over fully supervised approaches.

Supplementary Figure S1. Comparison of different annotation and map generation methods.

Supplementary Figure S2. Illustration of the localization ambiguity.

Supplementary Figure S3. Illustration of the optimal solution and the uniqueness of the proposed method.

Supplementary Figure S4. Typical visualization of the localization results on a human crowd using 10% of point annotations.

Supplementary Table S1. The comparison of different losses between density-map-based approaches and our method.

Supplementary Table S2. The summary of different methods related to this study.

Supplementary Table S3. The structure and split of each dataset used in this paper.

Supplementary Table S4. Benchmark of models: parameters, size, and methodology

Supplementary Note S1. Difference between density-map approaches and our method

The difference in loss functions between traditional density map-based approaches and the proposed method was shown in Supplementary Table S1. The density map-based approach outputs a predicted density map, while our method directly outputs a predicted location map $\hat{\mathcal{P}}$, as shown in the second row. The comparison, in simplified form, of a loss function is also shown; density map-based approaches had only a very limited effect on object localization.

Supplementary Note S2. Brief description of localization ambiguity

To reduce annotation costs, only partial instances in each training image are labeled with central point annotations. Thereafter, if a large kernel covers two or more targets while only one of them is labeled, the kernel may lead to localization ambiguity, which is shown in Supplementary Figure S1. It can be found that some entries are incorrectly calculated.

In this case, false count values are mixed into the redundant count map and thus appear in positive-unlabeled (PU) learning as positive samples that are lower than the ground truth. Therefore, false counts in redundant count maps would mislead the generation of predicted location maps, which tend to provide fewer local maxima than ground truth, especially in instance-dense regions.

Supplementary Note S3. Improve localization performance by pseudo-labeling

In the training phase, each image in the dataset is randomly and partially annotated with a fixed number of instances to simulate real application scenarios. For its corresponding redundant count map, all the area would belong to the unlabeled data except for the provided positive annotation. After the PU learning procedure, we exploit the strategy of pseudo-labeling to further improve localization performance in the unlabeled region.

In general, the location of each target can be obtained by heuristically searching for local maxima in the predicted location map, which is a natural way to select potential positives from the unlabeled region. However, false positives among the local maxima would definitely deteriorate the training process. Therefore, to adaptively categorize the unlabeled region into positive blocks and negative background, we adopt an unsupervised BMM to model the negative loss of the unlabeled region, which can be quantified by the element-wise distance between the predicted C_{pre} and the supplied count map:

$$\hat{C}_{gt}, \ \textbf{i.e.} \quad l_{i,j} = \left\{ \left| C_{pre}^{i,j} \right|^2 \middle| \hat{C}_{gt}{}^{i,j} = 0 \right\} \qquad \text{(S1)}$$

The subsequent steps in the pseudo-labeling design are shown in Figure 3. It is worth noting that we do not directly utilize the pseudo-labels of each unlabeled element classified by the BMM model, as counting noises can cause false positives. Instead, we select the top-ranked (first *N*) local maxima from the predicted location map as positive candidates. *N* indicates the number of targets and can be calculated from the sum of the predicted location map. Subsequently, the posterior probability of each candidate is further calculated to decide which one can be assigned positive labels.

In other words, if it is not a local maximum in the predicted location map, the corresponding block in the count map will be assigned negative labels. For instance, as shown in Figure 3, the blue square in the bottom middle of the predicted count map was assigned a predicted negative instead of a predicted positive, even if its posterior probability is higher than 0.6. A higher threshold would have yielded even purer positives, but at the cost of too few samples for learning. This pseudo-labeling strategy can

effectively reduce false positives and enhance the relationship between a target and a local maximum.

Supplementary Note S4. A Detailed illustration for the dataset split

The structure and split of each dataset used in this study are shown in Supplementary Table S1. The first 32 images and the subsequent 8 images in the Honeybee and Fish datasets are utilized for the training and validation samples, respectively. Colorectal adenocarcinoma (CA) cells and Modified Bone Marrow (MBM) datasets were randomly divided into training, validation, and test datasets with an approximate ratio of 2:1:2. For ShanghaiTech Part B, we randomly extracted 80 images from the training dataset provided as validation samples.

Supplementary Note S5. Theoretical derivation for the uniqueness of our method

We will prove the uniqueness of the optimal solution for the minimization problem of the following loss.

$$\min_{\Theta}|\mathcal{K} * \psi(\mathcal{J}, \Theta) - \mathcal{K} * \mathcal{P}| \qquad (S2)$$

where $\mathcal{J}$ and $\mathcal{P}$ denote a training image and its binary location map. $\psi(\mathcal{J}, \Theta)$ and $|\cdot|$ denote the output of a CNN with parameters Θ and a type of distance measure. $\mathcal{K}$ represents a kernel and $*$ is a convolution operator.

Proof. Since convolution is equivalent to a linear transformation, the minimization of Equation S2 can be rewritten as follows:

$$\min_{\Theta}\left|\mathbf{A}vec\big(\psi(\mathcal{J}, \Theta)\big) - \mathbf{A}vec(\mathcal{P})\right| \qquad (S3)$$

where **A** is the linear transformation, function *vec* $(\cdot)$ vectorizes the maps. By padding each image and its location map with $S_{\mathcal{K}} - 1$ columns/rows of *zero* on their **left/top** side, where $S_{\mathcal{K}}$ suggests the kernel size, **A** shapes **a lower triangular matrix**, as shown in the Supplementary Figure S2(a). Therefore, **A** has full rank and thus the unique optimal solution for $\psi(\mathcal{J}, \Theta)$ is $\mathcal{P}$.

Different kernels (all "1"/Gaussian) lead to different maps (redundant count map/density map). However, we use all "1" kernels instead of Gaussian kernels for two reasons. First, a Gaussian kernel will inevitably introduce an additional scaling factor σ (different from the kernel size). This scaling factor σ determines the width of the Gaussian function and controls the spread or smoothness of the kernel. Second, an all “1” kernel is equivalent to a Gaussian kernel with a very large σ, while the condition number of **A** decreases with the growth of σ and converges to a fixed value. It was shown in the Supplementary Figure S2(b), suggesting that all "1" kernel achieves a better robustness against the variation and label ambiguities in $\mathcal{P}$.

Supplementary Note S6. The benefits of the proposed method over fully supervised approaches

This partially supervised LFE framework offers several potential benefits over fully supervised approaches. A key benefit is that it allows for a more efficient use of labeled data. With a fully supervised approach, all data must be labeled, which can be time-consuming and costly. However, in this study, only a subset of the data needs to be labeled with point annotation instead of traditional block, which can significantly reduce the labeling effort. This can be particularly beneficial in situations where labeled data is scarce or expensive to obtain.

Another advantage of the LFE framework is that it can improve model generalization. Fully supervised approaches often rely on a limited amount of labeled data to train the model, which can lead to overfitting. By using unlabeled data in the training process, the LFE model generalizes better to unseen data. In addition, the partially supervised LFE framework can be more flexible and adaptable to changing data conditions. In cases where labeled data changes over time or becomes outdated, the model can still be updated and retrained using the partial supervision framework.

Both positive examples and unlabeled data are first fed into an encoder-decoder network, which outputs a predicted location map of a small object. Afterward, a predicted count map is yielded from the predicted location map and then shapes the PU loss for the PU learning procedure. The best model in the PU learning process acts as the initialization of the subsequent pseudo-labeling process, in which the negative loss of the unlabeled region in training images is first fitted as a beta mixture model (BMM), and then pseudo-labels are produced to conduct the ordinary binary classification.

Overall, the LFE framework offers several potential benefits compared to fully supervised approaches. Leveraging unlabeled data and improving model generalization can lead to more accurate predictions and better performance in real-world applications. Moreover, its flexibility and adaptability make it a valuable tool for continuous improvement and adaptation to new data, which can be critical in dynamic environments.

Supplementary Figure

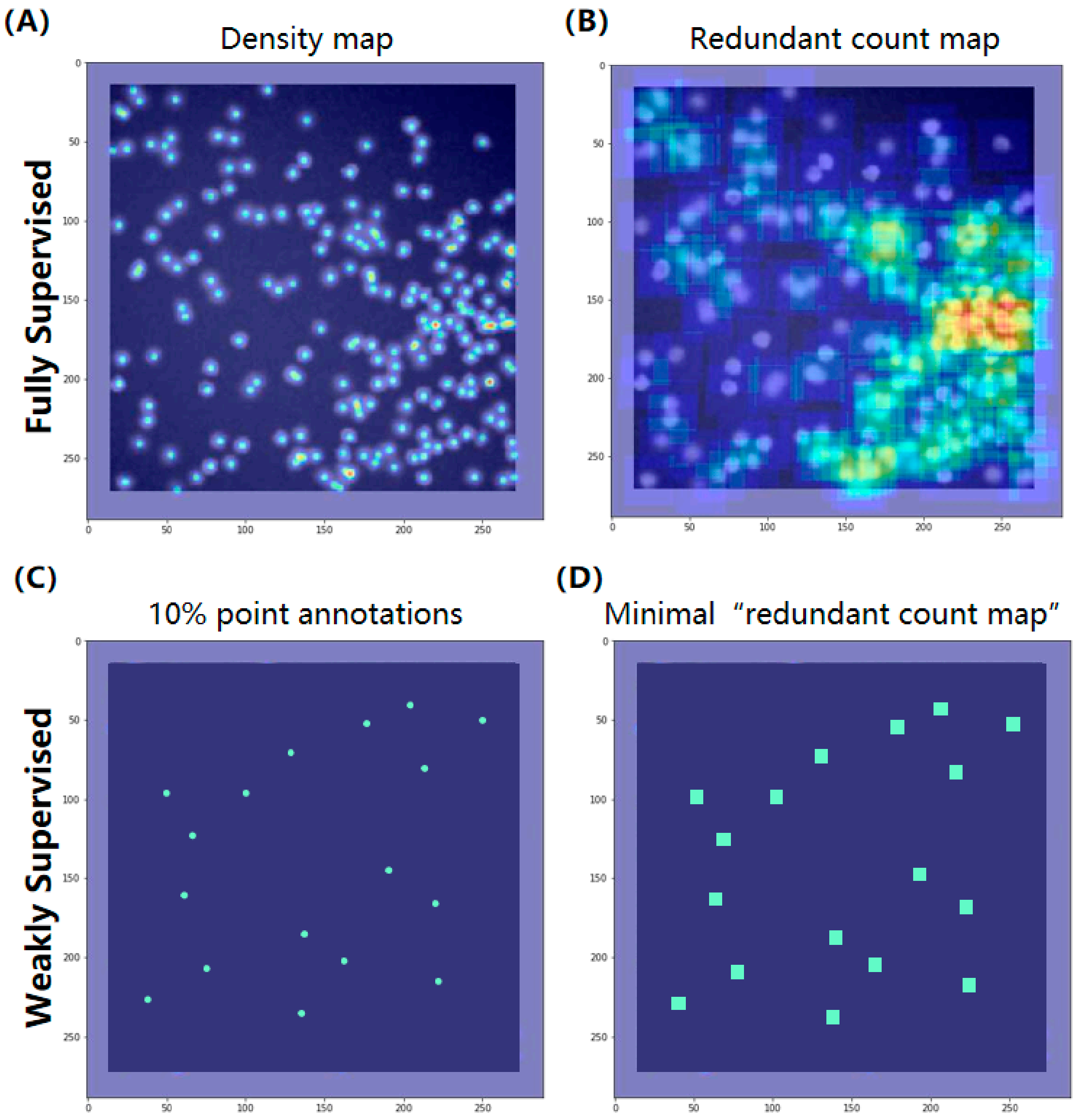

Figure S1: Comparison of different annotation and map generation methods. (A) A fully supervised density map generated using a Gaussian kernel. (B) A fully supervised count map generated using a square kernel. (C) Representative sparse annotations (10% of points) used in our weakly supervised approach, significantly reducing labeling effort and difficulty. (D) The proposed minimal "redundant count map" generated from these incomplete annotations.

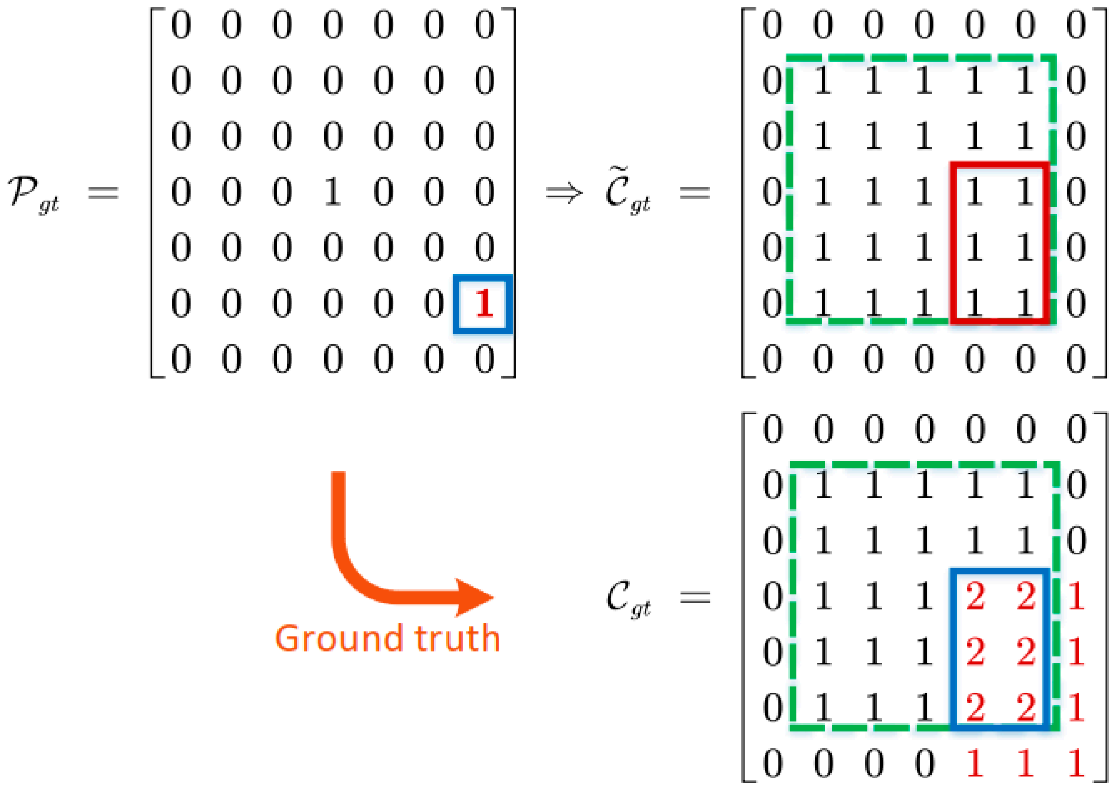

Figure S2. Illustration of the localization ambiguity. A kernel of 5×5 is adopted in the location map $\mathcal{P}_{gt}$, in which the red denotes an instance without point annotation. The elements surrounded by the green dotted square in $\hat{\mathcal{C}}_{gt}$ act as positive samples in the PU learning process while $\mathcal{C}_{gt}$ represent the true redundant count map. It can be found that the bottom right entries in the green square are two, but are wrongly computed in $\hat{\mathcal{C}}_{gt}$.

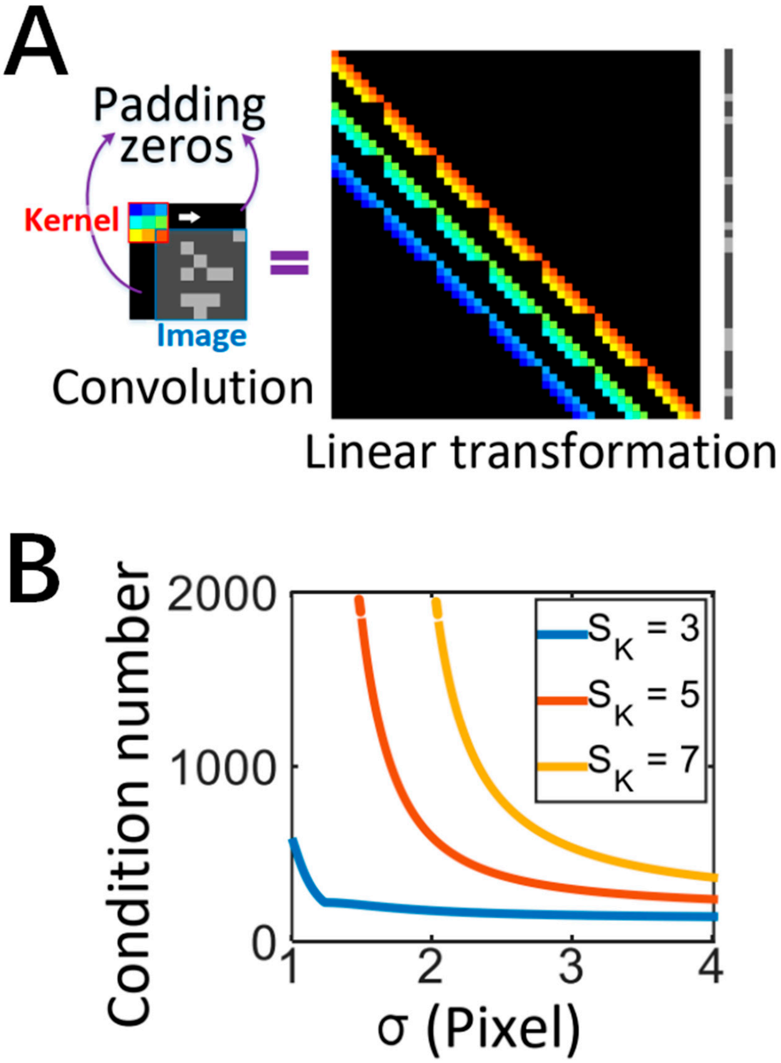

Figure S3. Illustration of the optimal solution and the uniqueness of the proposed method. (A) The convolution equivalent to a linear transformation forms a lower triangular matrix. (B) The condition number of **A** (linear transformation) decreases as the scaling factor σ grows and converges to a fixed value.

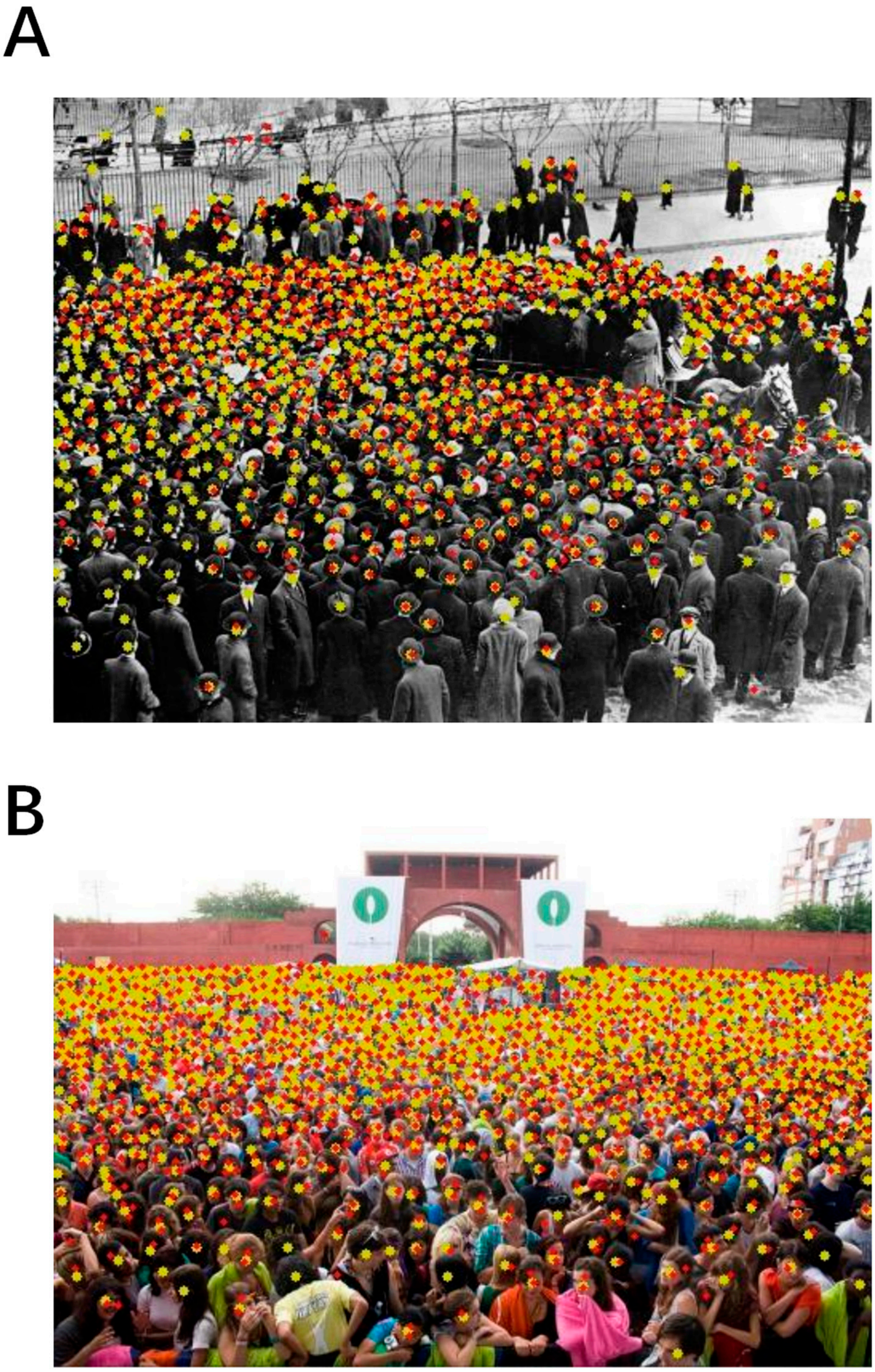

Figure S4. Typical visualization of the localization results on a human crowd using 10% of point annotations. The yellow circles and the red dots represent the true and the predicted centers of small instances, respectively.

Supplementary Table

Table S1. The comparison of different losses between density-map-based approaches and our method

Methods	Simplified form of loss	Predicted output	Counting function	Localization function
Density-map approach	$\min_{\Theta}\lvert\psi(\mathcal{J},\Theta)-\mathcal{D}\rvert$	Density map	Yes	Limited
Our method	$\min_{\Theta}\lvert\mathcal{K}*\psi(\mathcal{J},\Theta)-\mathcal{K}*\mathcal{P}\rvert$	Location map	Yes	Yes

Note: $\mathcal{J}$ and $\mathcal{P}$ denote a training image and its binary location map (1 for object centers, 0 for the rest region). $\psi(\mathcal{J},\Theta)$ and $\lvert\cdot\rvert$ denote the output of a CNN with parameters Θ and a type of distance measure. $\mathcal{D}$ denotes the corresponding pre-constructed density map. $\mathcal{K}$ represents a kernel and $*$ is a convolution operator.

Table S2. A summary of the different methods related to this study

Method	Brief summary	Limitations	Method Type
**SIDIP**	Uses density map estimation with 2D integer programming for small instance detection, incorporating global count constraints	High computational complexity; performance degradation in crowded scenes; requires full point annotations	Fully supervised density-map regressors
**SSAE**	Unsupervised learning approach using a stacked sparse autoencoder to learn high-level features from pixel intensities for nucleus detection	Long training time; limited handling of appearance variations; may produce blurry output maps	Unsupervised learning
**SR-CNN**	CNN-based structured regression model that outputs proximity maps encoding topological information for cell detection	Requires extensive point annotations; sensitive to annotation quality	Fully supervised density-map regressors
**SC-CNN**	Introduces spatial constraint regression layer that forces high probability values at nucleus centers, avoiding segmentation steps	Poor performance on overlapping cells; requires precise center point annotations	Fully supervised density-map regressors
**SFCN-OPI**	Sibling fully convolutional network unifying detection and classification through objectness prior interaction	Complex architecture with high resource demands; requires large annotated datasets	Supervised density-map regressors
**Count-ception**	FCN-based counting method using redundant counting for improved robustness (inferred from common usage)	Limited localization precision; requires dense annotations; not detailed in provided documents	Fully supervised density-map regressors
**SSL-LIR (our previous work)**	Strongly supervised LIRNet using local integral regression for nucleus detection with optimized counting and localization	Requires complete point annotations; high annotation cost; sensitive to data distribution changes	Fully supervised density-map regressors
**WSL-LIR** (our previous work)	Weakly supervised LIRNet using only patch-level count annotations, significantly reducing annotation cost by 74.3%	Performance drops with sparse annotations; relies on accurate patch-level labels; may miss small objects	Weakly supervised count-map regressors
**PU-based methods**	General category of positive-unlabeled learning methods that learn from positive and unlabeled data to reduce annotation costs	Prone to label noise; requires strategies to handle false negatives in unlabeled data	Weakly supervised learning
**LFE** (this paper)	Positive-unlabeled learning approach, learning from limited point annotations using minimal redundant count maps for small object localization	Parameters require tuning; sensitive to initial annotation quality	Weakly supervised location map and PU-based methods

Table S3. The structure and the split of each dataset used in this paper

Dataset	Resolution （W×H）	Number of images	Dataset split （training/validate/test）	Number of targets/images	Number of point annotation examples
Honeybee	640×480	118	32/ 8/ 78	28 ± 6	15, 10, 5, 3
Fish	300×410	129	32/ 8/ 89	56 ± 9	25, 15, 10, 5
CA cells	500×500	100	40/ 20/ 40	297 ± 217	100, 50, 25, 10
MBM cells	600×600	44	18/ 8/ 18	126 ± 33	50, 25, 10, 5
ShanghaiTech Part B	1024×768	716	320/ 80/ 316	123 ± 94	100, 50, 25, 12

Table S4. Benchmark of models: parameters, size, and methodology

**Method**	**Model parameters**	**Model Size**	**Method Type**
SSAE	1.48 M	5.64 MB	Unsupervised Learning
SR-CNN	1.225 M	4.78 MB	Strong Supervision
SFCN-OPI	13–22 M	50-90 MB	Strong Supervision
Count-ception	27.16 M	103.9 MB	Strong Supervision
SSL-LIR	2.65 M	10.1 MB	Strong Supervision
WSL-LIR	2.65 M	10.1 MB	Weak Supervision
LFE (this study)	1.55 M	6.3 MB	Weak Supervision
